# Strategies for the detection and prevention of human cytomegalovirus from pregnancy to the neonatal period

**DOI:** 10.3389/fcimb.2026.1965954

**Published:** 2026-10-09

**Authors:** Pei Hu, Wenjie Chen, Ying Zhou, Mengyao Yu, Yuexinzi Jin, Xi Chen, Jiexin Zhang, Jin Zhou

**Affiliations:** 1 Medical Laboratory Department, Gaoyou People’s Hospital, Gaoyou, China; 2 Department of Laboratory Medicine, the First Affiliated Hospital with Nanjing Medical University, Nanjing, China; 3 Department of STD/AIDS Control and Prevention, Jiangsu Provincial Center for Disease Control and Prevention, Nanjing, China; 4 Department of Clinical Laboratory, Children’s Hospital of Nanjing Medical University, Nanjing, China

**Keywords:** biomarkers, congenital infection, detection method, human cytomegalovirus, prevention strategy

## Abstract

Human cytomegalovirus (HCMV) is a major cause of congenital infection worldwide. Maternal infection with HCMV during pregnancy may lead to adverse fetal outcomes and long-term sequelae of congenital infection. In this review, we summarized three specimen sources, their corresponding valid indicators, and mainstream detection methods. Prevention strategies administered based on the results of these tests aim to reduce the risks of maternal infection and maternal-fetal transmission and the development of complications following congenital infection through behavioral and hygiene-related interventions, vaccination, and hyperimmune globulin and antiviral agent administration. In this review, we presented a flowchart for full-process HCMV infection management, which covers three levels of prevention nodes, confirmatory tests at each level, and corresponding interventional approaches. Future efforts require support from the funding, institutional, and educational sectors to accelerate the development of sensitive and inexpensive methods for the rapid identification of potential biomarkers.

## 1 Introduction

Cytomegalovirus (CMV), which belongs to the β-herpesvirus subfamily, is also known as human herpesvirus 5 because of its strict species specificity to only humans (Baasch et al., 2021; Kutle et al., 2023; Jia et al., 2025). Human cytomegalovirus (HCMV) is among the eight known double-stranded DNA herpesviruses that cause chronic or latent infections in humans (Atyeo et al., 2024). It is an opportunistic pathogen, and primary infection mostly occurs during infancy and early childhood (Yu et al., 2023; Kim et al., 2025). The seroprevalence of HCMV varies by socioeconomic background (Wang and Zhao, 2020). In countries with high maternal HCMV seroprevalence (>95%), the birth prevalence of congenital CMV (cCMV) infection is correspondingly high (>1%) (Leruez-Ville et al., 2017; Duri et al., 2021). HCMV can be transmitted through bodily fluids (such as blood, saliva, and urine), organ transplantation, and vertical transmission (Zhang et al., 2024). Clinical manifestations of HCMV infection may develop in immunocompromised individuals, and the virus can persist in lifelong latency in individuals with normal immune function (Khandazhinskaya et al., 2021; Yang et al., 2022; Otero et al., 2023; Schlaeffer-Yosef and Nesher, 2025).

## 2 Methods

This narrative review synthesizes literature primarily sourced from the PubMed database, with a search cutoff date of July 15, 2025. The reproducible search strategy employed the following string: (cytomegalovirus OR CMV OR human cytomegalovirus) AND (pregnancy OR gestation OR maternal OR congenital OR primary infection OR screening OR diagnosis OR prevention OR vaccine OR prognosis). The search was restricted to publications from January 2015 to July 2025, specifically targeting randomized controlled trials (RCTs), meta-analyses, systematic reviews, and clinical trials.

Eligible studies included: 1) peer-reviewed, English-language original research, systematic reviews, meta-analyses, and clinical consensus guidelines focusing on HCMV infection in pregnancy. Key topics encompassed maternal infection, cCMV diagnosis, neonatal detection, prevention strategies, and vaccine development; 2) publications reporting diagnostic test performance, clinical management data, intervention efficacy, or safety outcomes; and 3) seminal landmark studies published prior to January 2015. These earlier studies were manually included only if they constituted foundational clinical evidence underpinning current practice and were frequently cited in contemporary consensus documents.

Studies were excluded based on the following criteria: 1) non-human experimental studies (animal models), case reports, conference abstracts lacking full-text publication, and editorials or commentaries devoid of original or synthesizable clinical evidence; and 2) publications focusing exclusively on HCMV in solid organ or hematopoietic stem cell transplant (HSCT) recipients that lacked relevance to pregnancy or congenital infection.

Study selection followed a two-stage process. First, titles and abstracts of retrieved records were screened against predefined eligibility criteria; records deemed potentially relevant proceeded to full-text assessment for final inclusion. To capture landmark foundational publications, we manually searched the reference lists of included systematic reviews, key primary studies, and clinical consensus guidelines. This supplementary step allowed for the inclusion of highly cited seminal works published prior to 2015 (e.g., Nigro et al., 2005). These older studies were retained as they provide pivotal data that established core concepts for prenatal HCMV interventional research, which were not captured by the automated 2015–2025 database query. Data extracted from eligible publications were narratively synthesized and organized thematically into biomarkers, detection approaches, and prevention strategies.

## 3 Maternal HCMV infection

Pregnant women are not immunocompromised; rather, they maintain a unique immunological and physiological state to ensure fetal tolerance, increasing their susceptibility to HCMV infection (Arora and Lakshmi, 2021). The estimated global seroprevalence of HCMV among women of reproductive age was 85% (Johnson et al., 2024). A systematic review encompassing 29 studies published from 2010 to 2020 demonstrated marked regional heterogeneity in HCMV-IgG seroprevalence among women of reproductive age: Europe (45.6%–95.7%), Japan (60.2%), Latin America (58.3%–94.5%), and North America (24.6%–81.0%) (Fowler et al., 2022). A recent large-scale Turkish cohort study of pregnant women reported that the seropositivity of CMV IgM and IgG was 1.9% and 98.6%, respectively (Tiğli et al., 2025). Maternal primary HCMV infection (MPI) refers to first-time infection in pregnant women with no pre-existing HCMV-specific IgG antibody, whereas maternal non-primary HCMV infection (MNPI) refers to active HCMV infection in pregnant women with pre-existing HCMV-IgG antibody due to exogenous reinfection by a different strain or reactivation of an endogenous latent strain (Leber, 2024). Sartori et al. demonstrated that seronegative women who conceive a second child within two years of their first pregnancy have a 6% risk of primary infection in early pregnancy, which is 19 times greater than that of the general population (Sartori et al., 2023). The main route of HCMV infection in pregnant women is close contact with toddlers, and the most common sources of infection are saliva and urine from infected toddlers (Khalil et al., 2025). The risk of cCMV infection increases with advancing gestational age in the context of MPI, which is lowest (21%–36.8%) during conception and early pregnancy and increases to 72% in mid-to-late pregnancy (Chatzakis et al., 2020; Buca et al., 2021; Plotogea et al., 2022). However, 20%–30% of infections during conception and early pregnancy, compared with only 1% of infections in mid-to-late pregnancy, cause fetal intrauterine growth restriction (Slots and Slots, 2024). In cases of MNPI, the rate of cCMV infection is 0.4%–6.1% (Lawrence, 2024). Both MPI and MNPI can lead to cCMV infection in fetuses and neonates, with vertical transmission rates of 30%–40% and 1%–2%, respectively (Messinger et al., 2020). The risk of sequelae at birth is comparable between these two groups (Maltezou et al., 2020). In a study conducted by Yamamoto et al. involving a study population with nearly universal CMV seroimmunity, evidence of infection with multiple CMV strains was found in 52.5% of mothers who gave birth to cCMV-infected neonates, compared with only 20% of the control group. These findings confirm that maternal reinfection with novel HCMV strains serves as a critical predisposing factor for cCMV infection (Yamamoto et al., 2010).

## 4 cCMV related sequelae

Approximately 0.3%–2.3% of newborns are affected by HCMV (Shang and Li, 2024). Notably, the disease burden attributable to cCMV infection far exceeds that attributable to other common congenital pediatric disorders, including Down syndrome, fetal alcohol syndrome, and spina bifida (Huang SN, et al, 2021). HCMV is associated with fetal intrauterine growth restriction, miscarriage, stillbirth, and neonatal death (Megli and Coyne, 2022). Approximately 10% of infected neonates present with clinical symptoms at birth; these symptoms may include low birth weight, purpura, jaundice, hepatosplenomegaly, retinitis, microcephaly, hearing impairment, and thrombocytopenia. Of these symptomatic neonates, more than 90% will develop long-term sequelae, and 10%–30% will not survive. Among asymptomatic neonates at birth, 10%–15% may have long-term neurological sequelae, including sensorineural hearing loss (SNHL) and neurodevelopmental disorders (Messinger et al., 2020; Pighi et al., 2024).

### 4.1 SNHL

cCMV infection is the leading cause of nonhereditary childhood SNHL (Rouse et al., 2022). It has been reported that 11%–50% of children with SNHL exhibit progressive hearing deterioration; 16%–23% of children with SNHL may present with fluctuating hearing loss, which can be either unilateral or bilateral (Pinninti et al., 2024). Furthermore, 33%–50% of SNHL cases occur after the neonatal period (so-called “late-onset SNHL”) (Pinninti and Boppana, 2022). Transient hearing loss during the critical period of language and speech development can impair the maturation of central auditory function (Choe et al., 2023).

### 4.2 Neurodevelopmental disorders

cCMV infection is also an important cause of severe neurodevelopmental disorders in children (Penner et al., 2021). cCMV-associated vestibular insufficiency (cCMV-VI), which involves gross motor developmental delay, often coexists with SNHL but may also occur independently (Leruez-Ville et al., 2020). Pinninti et al. performed comprehensive assessments of vestibular function, gaze function, and balance in 40 children with asymptomatic cCMV infection who were identified on newborn screening and reported a 17.5% incidence of hearing loss and a 45% incidence of cCMV-VI (Pinninti et al., 2024). In a large cohort study, Faure-Bardon et al. reported that SNHL and neurological sequelae were present only in neonates born to mothers with MPI during early pregnancy (Faure-Bardon et al., 2019). The authors recommended auditory and specialized neurologic follow-up only in cases of MPI during the first trimester.

## 5 Maternal HCMV-related markers

### 5.1 Blood samples

HCMV-IgG seroconversion, the presence of HCMV-IgM, a low HCMV-IgG avidity index (AI), and the presence of DNAemia constitute the primary criteria for the diagnosis of MPI during pregnancy to determine the timing of MPI and assess the risk of fetal infection (Sarasini et al., 2021) (**Table 1**).

**Table 1:** Summary of specimen-based methods for HCMV infection.

Specimen type	Diagnostic method	Optimal timing	Clinical role	Diagnostic performance	Key limitations
Maternal peripheral blood serum	HCMV-IgG	Initial first-trimester prenatal visit	Identify susceptibility status; IgG seroconversion confirms MPI	Seroconversion is highly specific for MPI; single IgG result cannot distinguish past v.s. recent infection	Requires paired 2-4-week follow-up specimens for seroconversion confirmation
Maternal peripheral blood serum	HCMV-IgM	Immediately upon suspected MPI	Indicator of recent infection; adjunct test only	High sensitivity on CLIA platforms with prolonged positivity; shorter-duration positivity on ELISA; moderate specificity	May occur during reactivation; EBV cross-reactivity exists; cannot diagnose MPI independently; substantial inter-platform variability
Maternal peripheral blood serum	HCMV-IgG AI	After IgG seroconversion	Distinguish primary infection within the preceding 3-4 months	Low avidity supports recent primary infection; a proportion of results fall into the grey zone	Not interpretable for samples with very low IgG titres; indeterminate grey-zone results; only for auxiliary diagnosis
Maternal whole blood/plasma	HCMV-DNA PCR	Within one month following MPI onset	Detect viremia and evaluate vertical-transmission risk	Positive viremia indicates elevated vertical-transmission risk; no universally agreed viral-load cut-offs	Persistent viremia may occur in non-primary infection; cannot differentiate MPI/MNPI; serves only as an adjunct biomarker
Maternal peripheral-blood leukocytes	PP65 antigenemia	Early phase of infection	Assess viral replication level	Lower sensitivity compared with PCR; good correlation with clinical symptoms in immunocompromised hosts	Lack of standardization; requires >1,000 cells/mL; limited clinical value in pregnancy
Maternal peripheral blood	IGRA	Any time after infection onset	Evaluate cellular-immune status; potential prognostic biomarker	100% sensitivity in transplant cohorts; predictive values ranging from 23%-46%	No consensus cut-offs for pregnant populations; investigational only, not for routine clinical practice
CVS	HCMV-DNA PCR	Gestational weeks 11-14	Evaluate placental infection in early pregnancy	High specificity and favourable negative predictive value	Invasive procedure; evidence requires further long-term follow-up validation; not recommended for routine prenatal diagnosis
Amniotic fluid	HCMV-DNA PCR	≥18-20 weeks gestation; ≥8 weeks after MPI onset	Reference-standard prenatal diagnosis for cCMV	Sensitivity 90%-95%; 5%-10% false-negative rate	High false-negative risk with overly early sampling; invasive procedure; cannot directly predict fetal prognosis
Fetal cord blood	Complete blood count, β_2_-microglobulin, HCMV-DNA PCR	After fetal infection is confirmed by amniotic-fluid testing	Prognostic risk-stratification for fetuses	Thrombocytopenia and high viral load suggest poor outcome; negative predictive value reaches 100% when combined with ultrasound	Invasive miscarriage-related risk; inconsistent cut-offs across studies; not intended for initial screening
Breast milk	HCMV-DNA PCR	Postpartum days 3-4 weeks	Evaluate breast-milk viral shedding; guide breastfeeding management for preterm infants	High proportion of positive results in early postpartum breast-milk samples	Cannot directly predict infant disease; only for risk-stratification purposes
Neonatal saliva	HCMV-DNA PCR	≤21 days after birth	First-line cCMV diagnostic test; FDA-cleared commercial assays available	Sensitivity 94.64%; saliva viral load >2.5 log copies/mL helps discriminate true- versus false-positive results	Contamination after breastfeeding may cause false-positive results; sampling ≥1 h post-feeding advised; positive results require confirmation using urine specimens
Neonatal urine	HCMV-DNA PCR	≤21 days after birth	Reference-standard test for cCMV diagnosis	High sensitivity and high specificity	Logistical challenges for neonatal urine collection
Neonatal DBS	HCMV-DNA PCR	Within the first week of life	Screening/Retrospective investigative tool	Sensitivity ranges 34.4%-100%; specificity 98.1%-99.0%	Negative result cannot rule out cCMV; low perinatal viral load in some neonates predisposes to false-negative findings; not recommended as first-line screening
DUC	HCMV-DNA PCR	Collected at delivery	Alternative retrospective archival specimen	Limited sample size and sparse clinical data	Investigational only; no standardized clinical workflow available

#### 5.1.1 HCMV DNA

HCMV DNA can be detected in maternal blood within one month after MPI (Huang et al., 2024). Although prolonged viremia during MPI has been reported to be associated with the risk of vertical transmission, the HCMV viral load (VL) can serve only as an adjunct to serological diagnosis (Gerna et al., 2021). A positive PCR result does not necessarily indicate a recent primary infection, given that viremia may remain persistently positive. Studies have reported that maternal HCMV viremia indicates a higher risk of HCMV vertical transmission: this risk increases by 5.7-, 6.5-, and 13.0-fold when viremia occurs in the first, second, and third trimesters, respectively (Huang et al., 2024). Therefore, the time required for blood specimen collection should be fully considered for viremia confirmation.

#### 5.1.2 HCMV-IgG

On the basis of the results of serum HCMV-IgG detection, women in early pregnancy are divided into an HCMV-seropositive group and an HCMV-seronegative group (Huang Y, et al, 2021). IgG seroconversion, which is confirmed by serum HCMV-IgG positivity 2–4 weeks after a previous negative HCMV-IgG result, is an essential diagnostic criterion for MPI (Gerna et al., 2021).

#### 5.1.3 HCMV-IgG avidity

HCMV-IgG avidity testing is an important tool for the diagnosis of MPI, since the HCMV-IgG AI typically increases over time; this index remains low during the first trimester after infection and gradually increases to high levels thereafter (Sarasini et al., 2021). HCMV-IgG avidity is classified into three levels: (1) low avidity, indicating primary infection within the past 3–4 months; (2) high avidity, which can rule out recent primary infection; and (3) intermediate avidity (also called “the gray zone”). The combination of a low HCMV-IgG AI and positive HCMV-IgM result strongly supports primary infection within the past 3–4 months (Manicklal et al., 2013). Gerna et al. suggested that antibody avidity testing should not be performed on serum specimens with low HCMV-IgG antibody levels (Gerna et al., 2021). Kitamura et al. investigated the cutoff value for the HCMV-IgM titer used in HCMV-IgG avidity testing for maternal screening (Kitamura et al., 2023). The data suggested that compared with testing all HCMV-IgM-positive pregnant women, performing avidity testing in only pregnant women with a high HCMV-IgM titer is more effective at identifying those with primary infection.

#### 5.1.4 HCMV-IgM

HCMV-IgM may persist for 9 to 12 months in 25% of individuals after primary infection. Viral reactivation can also elicit HCMV-IgM production, allowing HCMV-IgM to be detected during nonprimary infection (Leber, 2024). HCMV-IgM cross-reactivity can occur with other viral infections, such as Epstein-Barr virus (EBV) infection, and nonspecific polyclonal immune stimulation may also result in the detection of HCMV-IgM (Périllaud-Dubois et al., 2022). Accordingly, the use of HCMV-IgM as a sole marker for MPI is associated with inconsistent sensitivity and suboptimal specificity. A study by Sarasini et al. investigating the kinetics of HCMV-IgM production using a chemiluminescent immunoassay (CLIA) and an enzyme-linked immunosorbent assay (ELISA) demonstrated that the choice of serological method (ELISA versus CLIA) is critical for the detection of HCMV-IgM (Sarasini et al., 2021). The results revealed that 62% of pregnant women remained HCMV-IgM positive by CLIA for more than 180 days after the onset of MPI, while only 13% were HCMV-IgM positive by ELISA.

#### 5.1.5 HCMV antigenemia

HCMV antigenemia refers to the presence of HCMV antigens, specifically phosphoprotein 65 (PP65), within peripheral blood leukocytes (PBLs) (Ljungman et al., 2017). Li et al. detected the PP65 antigen in PBLs within the first week after HCMV infection using immunohistochemistry or immunofluorescence because PP65 antigenemia is usually elevated during this period (Li et al., 2023). Jerry Teng et al. reported that although the sensitivity of antigenemia testing is lower than that of PCR, the results of antigenemia testing more closely correlate with clinical symptoms in immunocompromised patients (Jerry Teng et al., 2021). However, the clinical utility of antigenemia testing for HCMV is limited in most laboratories due to a lack of standardization and the requirement for a high number of PBLs (>1,000 cells/mL). Consequently, this assay is primarily restricted to research settings involving immunocompromised individuals and is not recommended for routine clinical application in pregnancy (Li et al., 2023).

#### 5.1.6 HCMV-specific T cells

Sequential monitoring of HCMV-specific T cells may provide potentially useful information for the management of HCMV infection and is considered an additional biomarker of HCMV DNA load (Ljungman et al., 2019). Immediate early protein 1 (IE-1) and PP65, which are encoded by HCMV, stimulate CD4^+^ and CD8^+^ T cells to release interferon-γ (IFN-γ), whose concentration can be used to quantify HCMV-specific immunity (Chemaly et al., 2020). The two most widely used IFN-γ release assays (IGRAs) are the ELISpot-based T-SPOT.CMV assay and the ELISA-based QuantiFERON-CMV assay (Annaloro et al., 2020). Gliga et al. compared T-SPOT.CMV and QuantiFERON-CMV assay results in liver transplant recipients to determine HCMV-specific cellular immunity (Gliga et al., 2021). In this study, the predictive values of T-SPOT.CMV and QuantiFERON-CMV were 46% and 23%, respectively, although both assays were 100% sensitive. While the T-SPOT.CMV assay has shown promising clinical value for predicting viral reactivation, sequential monitoring of HCMV-specific T-cell responses via IGRAs has demonstrated predictive utility primarily in transplant cohorts; its application in obstetrics remains unproven. Because optimal cut-off values and clinical predictive performance have not been adequately validated for pregnant women or fetuses, these cellular immune assays currently serve only as investigational adjunct biomarkers and are not recommended for routine clinical use in obstetric populations.

### 5.2 Breast milk

HCMV reactivation is estimated to occur in up to 96% of HCMV-seropositive women, and viral DNA is shed from breast milk during the postpartum period (Bateman et al., 2021). A retrospective analysis by Hernandez-Alvarado et al. revealed an overall transmission rate of 13.6% when blood samples from infants exposed to HCMV-positive breast milk were analyzed (Hernandez-Alvarado et al., 2021). A literature review and experience from an Italian hospital revealed that postnatally acquired HCMV infection is mostly asymptomatic in term infants (Garofoli et al., 2021). In contrast, infants with a very low birth weight (VLBW, <1,500 g) and an extremely low birth weight (ELBW, <1,000 g) may present with severe clinical manifestations. A systematic review by Kurath et al. noted that up to 34.5% of infected preterm infants may develop clinical manifestations of HCMV, including liver disease, neutropenia, thrombocytopenia, and a sepsis-like syndrome (Kurath et al., 2010). A meta-analysis revealed that among VLBW infants born to HCMV-seropositive mothers, approximately 19% of those fed untreated breast milk acquired postnatal HCMV infection, of which 4% developed an HCMV-related sepsis-like syndrome (Lanzieri et al., 2013). In a prospective study, 45.9% of breast milk was positive for HCMV DNA within three days postpartum, and the VL was comparable to that at four weeks postpartum (Romero-Gómez et al., 2015). In addition, PCR can be used to effectively identify mothers with a high HCMV DNA load in their breast milk and help determine whether frozen breast milk should be fed to preterm infants with <28 weeks of gestation. Liu et al. combined multienzyme isothermal rapid amplification (MIRA) with a lateral flow dipstick (LFD) to establish a simple, rapid, and cost-effective assay suitable for detecting the conserved HCMV sequence UL83 in breast milk (Liu et al., 2024). With a 12-minute reaction time and an extra 10 minutes required to read the results, home-based self-testing has become feasible. Nevertheless, the validation of these assays has been confined to laboratory settings. Due to the absence of data from large-scale clinical cohorts, they have yet to obtain regulatory approval for clinical use.

## 6 Fetal specimens for HCMV detection

Specimen types derived from mothers and fetuses are diverse in terms of suspected infection stage and the timing of sample collection (**Table 1**).

### 6.1 Chorionic villus sampling

Maternal HCMV viremia in the first trimester leads to the viral invasion of syncytiotrophoblasts and cytotrophoblasts prior to or concomitant with fetal infection (Pereira, 2018). A feasibility study by Faure-Bardon et al. demonstrated that following HCMV MPI in the first trimester, placental infection can be diagnosed in the first trimester using CVS with PCR amplification of viral genomes in chorionic villi, yielding high specificity and a high negative predictive value (Faure-Bardon et al., 2021). The Faure-Bardon team proposed that a negative HCMV PCR result in trophoblast cells after 12 weeks of gestation can be used to exclude HCMV-related embryopathy that leads to sequelae. In a large RCT by Malan et al., the risk of miscarriage associated with the invasive procedure of CVS was <1‰ (Malan et al., 2018). However, long-term follow-up is needed to confirm these results, and it is not recommended for routine prenatal diagnosis.

### 6.2 Amniotic fluid sampling

Detecting HCMV DNA in amniotic fluid remains the reference standard for diagnosing prenatal intrauterine infection (Prabhu et al., 2021). The timing of amniocentesis is critical. The International Society of Ultrasound in Obstetrics and Gynecology recommends that amniocentesis should be delayed until more than 18–20 weeks of gestation and more than 8 weeks after MPI (Khalil et al., 2020). The rate of a false-negative amniocentesis result was higher when samples were obtained less than 8 weeks after MPI (Dinsmoor et al., 2022). The sensitivity of prenatal diagnosis by PCR in amniotic fluid has been reported to be between 90% and 95%. The false-negative prenatal diagnosis rate of 5%–10% (i.e., neonates that were born infected following a negative prenatal diagnosis) can be explained by transplacental passage of the virus later than 6 to 8 weeks after MPI (Revello et al., 2008; Leruez-Ville and Ville, 2017). By pooling data from seven observational studies, Chatzakis et al. reported no severe SNHL and/or neurodevelopmental impairment at follow-up in children born to pregnant women with HCMV infection during pregnancy and a negative amniocentesis result (Chatzakis et al., 2023). Thus, a negative HCMV PCR result in amniotic fluid obtained via appropriately-timed amniocentesis constitutes a strong and reassuring finding for fetal prognosis (Leruez-Ville et al., 2024).

### 6.3 Cordocentesis sampling

Once fetal HCMV infection is confirmed by amniocentesis, cordocentesis can be used to acquire blood samples for prognostic risk stratification. Given its high-risk invasive nature, it is unsuitable for initial screening. Additionally, cut-off values for platelet count, β_2_-microglobulin, and VL lack standardization across clinical centers. Several fetal blood parameters, particularly fetal thrombocytopenia, are associated with poor prognosis in fetuses with cCMV infection (Fabbri et al., 2011). A study by Faure-Bardon et al. demonstrated that the coexistence of thrombocytopenia and mild sonographic signs of infection during the second trimester is associated with a 60% risk of fetal symptoms at birth or termination of pregnancy due to severe brain lesions (Faure-Bardon et al., 2020). A retrospective analysis by Leruez-Ville et al. revealed that fetal blood parameters (e.g., fetal platelet count and the fetal blood HCMV DNA load), along with ultrasound findings, were independent prognostic factors for symptoms at birth (Leruez-Ville et al., 2016). Compared with VL in the amniotic fluid, fetal blood parameters had superior predictive value for symptomatic status at birth. The negative predictive value of ultrasound for symptomatic status at birth or termination was 93%, that of ultrasound plus VL in the amniotic fluid was 95%, and that of ultrasound plus fetal blood parameters reached 100%. Multiple fetal blood biomarkers have been reported to correlate with symptomatic cCMV outcome. Fabbri et al. identified optimal receiver operating characteristic-derived cut-offs of thrombocytopenia (<50,000/mm³) and elevated β_2_-microglobulin (>11.5 mg/L), whereas Leruez-Ville et al. used thresholds of fetal platelet count ≤114,000/mm³ and fetal-blood VL ≥4.93 log_10_ IU/mL for risk stratification (Fabbri et al., 2011; Leruez-Ville et al., 2016). Hawkins-Villarreal et al. assessed the predictive value of fetal blood parameters for severe cerebral anomalies according to gestational age and reported that platelet count and serum gamma glutamyl transferase level were significantly correlated with severe brain damage in cCMV-infected fetuses (Hawkins-Villarreal et al., 2019). In severely affected fetuses, blood parameters vary with gestational age and gradually approach the normal reference range during the third trimester, which may reduce their predictive value for brain damage.

## 7 Neonatal HCMV detection

The detection of HCMV DNA or virus isolation from neonatal saliva or urine specimens collected within the first 2 to 3 weeks of life constitutes the definitive diagnostic criterion (de-facto gold standard) for confirming cCMV infection (Razonable et al., 2020). Viral culture includes both traditional tissue tube culture (the empty spot assay) and vial culture. Although viral culture allows the detection of early HCMV antigens before the appearance of characteristic cytopathic effects in tissue tube culture and has a short turnaround time (48 hours), its diagnostic sensitivity is significantly lower than that of antigen detection and molecular assays and remains insufficient for clinical application (**Table 1**).

### 7.1 Saliva and urine

Neonates with cCMV infection exhibit high VLs in saliva and urine. To distinguish congenital infection from postnatal infection, specimens should be collected and tested within 2–3 weeks after birth (Gantt et al., 2020).

Saliva specimens are collected by oral swabbing, which is well accepted by parents. Exler et al. performed saliva PCR in 133 neonates and reported 10 false-positive results indicating VLs that were significantly lower than those in true-positive samples (Exler et al., 2019). These false-positive results were due to genital tract contamination or recent breastfeeding (Razonable et al., 2020). Tan et al. suggested that saliva samples should be collected ≥1 hour after breastfeeding to avoid confusion with low-level false-positive results (Tan et al., 2023). Nevertheless, a positive result for saliva detection must be confirmed by a second round of testing within 3 weeks of birth, and urine is typically chosen as an alternative (Huang Y. et al., 2021). On the basis of the results of a 3-year study, Eventov-Friedman et al. established a saliva VL cutoff of 2.5 log DNA copies/mL, which can reliably distinguish between true-positive and false-positive saliva results with a sensitivity of 98.2%, a positive predictive value of 86.2%, and a negative predictive value of 96.8% (Eventov-Friedman et al., 2019).

Although the collection procedure is more challenging, PCR testing of urine remains the gold standard for diagnosing cCMV in neonates within 21 days of life (Dunn et al., 2023). Lawrence et al. reported that viruria is generally prolonged, and no association between long-term growth retardation or cognitive impairment and the duration of viral excretion has been identified (Lawrence, 2024). However, a greater proportion of children whose viral excretion duration was <4 years developed SNHL and progressive SNHL than those whose viral excretion duration was >4 years.

Only two qualitative methods have been approved by the U.S. Food and Drug Administration (FDA) for the diagnosis of cCMV in neonates: the Alethia CMV DNA amplification assay and the Simplexa cCMV Direct real-time PCR assay (Gantt et al., 2020; Dunn et al., 2023). The Simplexa cCMV Direct assay is validated for both saliva and urine neonatal specimens, whereas the Alethia CMV assay is FDA-cleared only for saliva swab testing. In a small retrospective study using archived specimens, the Simplexa cCMV Direct assay showed a combined 94.64% sensitivity and 98.04% specificity with undiluted urine and saliva swab specimens using the FDA-cleared assay definition (a C_T_ positivity cutoff ≤ 37.5) (Fernholz et al., 2023).

### 7.2 Dried blood spot

A positive urine or saliva PCR result beyond 3 weeks of life cannot reliably distinguish cCMV infection from perinatal infection (Auriti et al., 2021). Perinatal infection is mostly acquired via exposure to infected maternal cervical secretions or breastfeeding and generally does not cause clinical disease or long-term sequelae. DBS samples are originally collected during the first week of life for routine newborn genetic and congenital disease screening. DBS PCR serves as a retrospective investigative tool for suspected cCMV infection (Pinninti and Boppana, 2022). Leruez-Ville et al. assessed the diagnostic performance of two DBS PCR assays for cCMV screening in a high-risk neonatal cohort (Leruez-Ville et al., 2011). The assays yielded sensitivity of 95.0%-100%, specificity of 98.1%-99.0%, positive predictive value of 94.1%-96.9%, and negative predictive value of 98.5%-100%. In a prospective observational study conducted by Chung et al., DBS PCR was performed on 1,374 neonates and had a sensitivity of at least 84% and a specificity of 99.9% (Chung et al., 2023). However, a negative DBS result does not rule out cCMV infection. In a large prospective HCMV screening study in newborns, Boppana et al. directly compared DBS real-time PCR with saliva culture and demonstrated that even the highly sensitive two-primer DBS real-time PCR assay may miss up to 80% of cCMV infections (observed sensitivity 34.4%, 95% CI 18.6%-53.2%) (Boppana et al., 2010). This high rate of false negatives may be attributed to the fact that not all neonates with cCMV infection have detectable HCMV DNA in their peripheral blood at birth. Moreover, DBS PCR performance differs substantially between high-risk and general newborn populations, indicating that high sensitivity reported in selected cohorts cannot be extrapolated to universal screening. According to the 2024 European Congenital Cytomegalovirus Infection Initiative consensus, a positive DBS PCR result serves only as preliminary screening evidence (Leruez-Ville et al., 2024). A definitive diagnosis must be established via HCMV PCR on neonatal urine or saliva specimens collected within the first three weeks of life. In a retrospective study, Del Valle Penella et al. reported that patients with true-positive DBS assay results had higher median blood HCMV levels than those with false-negative results did (Del Valle Penella et al., 2023). Limaye et al. reported that at clinically relevant VLs, HCMV loads in DBS were generally comparable to those in plasma samples, and changes in HCMV load during treatment were similar between the two specimen types, indicating that DBS assays may also be useful in monitoring therapeutic responses (Limaye et al., 2013).

### 7.3 Blood

Neonatal blood is not recommended as the primary specimen for diagnosing cCMV infection, since viremia is detectable at birth in only 10%–20% of cCMV-infected neonates (Leber, 2024). Reyes et al. compared HCMV loads in various specimens from cCMV-infected neonates at birth and reported that in both symptomatic and asymptomatic patients, peripheral blood VLs were lower than saliva and urine VLs (Reyes et al., 2020). High VLs in neonatal blood are associated mainly with SNHL (Krstanović et al., 2021). A collaborative research group evaluating the utility of HCMV antivirals in symptomatic infections suggested that the VL at baseline or during treatment should not be used as a marker for assessing the efficacy of cCMV therapy, nor should it guide treatment decisions, including the initiation or discontinuation of therapy (Leber, 2024). Medoro et al. investigated the association between peripheral T-cell responses and neurodevelopmental outcomes in 30 neonates with cCMV infection and 15 uninfected neonates and reported that the absence of a CD8^+^ T-cell response to cCMV infection *in utero* was linked to neurodevelopmental delay, regardless of symptom status at birth (Medoro et al., 2024). However, these findings warrant further validation in a larger cohort of neonates with cCMV infection.

### 7.4 Dried umbilical cord

Koyano et al. detected HCMV DNA in DUC specimens from twenty children to systematically evaluate the impact of cCMV infection on developmental delay (Koyano et al., 2009). A total of five children were HCMV DNA positive and asymptomatic at birth, among whom four had intracranial calcifications. Asymptomatic cCMV infection is an important cause of developmental disability. Reyes et al. studied DUC specimens from sixteen infants with cCMV infection and detected HCMV DNA in 3 of the infants (Reyes et al., 2020). Two infants were DBS negative, and one had a higher VL in DUC than in DBS specimens. The authors emphasized that stored DUC specimens represent a viable alternative for the retrospective diagnosis of cCMV infection, especially in cases where DBS assays produce false-negative results. Nevertheless, the application of DUC specimens is currently restricted to research settings, and a standardized clinical protocol has yet to be developed.

## 8 Prevention strategies

To reduce maternal infection, mother-to-fetus transmission, and cCMV-related sequelae, a three-step prevention strategy can be referenced and used (**Table 2**).

**Table 2:** Prophylactic and therapeutic strategies for HCMV infection.

Intervention	Target population	Intended outcome	Highest-level of supporting evidence	Current clinical status (evidence cutoff: July 2025)	Major limitations
Hygiene-related behavioural counselling	Seronegative pregnant women/preconception women at risk of HCMV exposure	Reduce risk of maternal primary HCMV infection	Controlled prospective intervention study	Recommended routine health-education measure in obstetric care	Effect size moderate; compliance with behavioural measures varies between individuals; cannot fully eliminate infection risk
HIG	Pregnant women with confirmed maternal primary HCMV infection (prior to confirmed fetal infection)	Reduce risk of intrauterine HCMV transmission	Multicenter double-blind RCT (negative primary outcome)	Investigational intervention; not recommended for routine clinical use; only permitted within prospective clinical trials	Early positive signals derived from non-randomized observational cohorts subject to selection bias; high-quality RCT failed to demonstrate clinical benefit; short in-vivo half-life requires repeated infusions; high cost
VCV (secondary-prevention, off-label)	Pregnant women with periconceptional/first-trimester MPI, before confirmation of fetal intrauterine infection	Reduce vertical transmission risk of HCMV	Randomized double-blind placebo-controlled trial + individual-patient-data meta-analysis	Conditional off-label consideration; specialist-led decision-making, mandatory risk-benefit assessment and written informed consent; no regulatory obstetric indication	Only effective mainly in viremia-positive MPI patients; no universal validated viral-load cut-off; safety data in later-trimester MPI remain limited
VCV (tertiary-prevention, investigational)	Pregnancies with already confirmed fetal intrauterine HCMV infection (positive amniotic-fluid HCMV-DNA PCR)	Improve fetal/neonatal clinical outcomes after established cCMV infection	Multicenter open-label single-arm phase II trial	Purely investigational; not for routine clinical application	Absence of randomized controlled trial validation; long-term maternal-fetal safety data remain insufficient; cannot be regarded as proven therapy
HCMV candidate vaccines (gB/MF59, V160, mRNA-1647, VBI-1501 etc.)	Seronegative adolescents/women of child-bearing age; modelled for infant vaccination	Prevent maternal primary HCMV infection, reduce population-level cCMV burden	Completed phase II RCTs (gB-MF59, mRNA-1647); mathematical modelling for infant-vaccination scenarios	No HCMV vaccine is currently licensed for human use; mRNA-1647 is undergoing a phase III RCT (NCT05085366)	gB-MF59 shows only ~50% waning protective efficacy; modelled childhood-vaccination benefits are theoretical simulation outputs without empirical clinical validation

### 8.1 First-step prevention

#### 8.1.1 Behavioral and hygiene-related interventions

The International Congenital HCMV Recommendations Group (ICCRG) recommends that all pregnant women receive education focused on HCMV-related diseases and prevention strategies (Rawlinson et al., 2017). Behavior-modification hygiene measures, including minimizing exposure to saliva and urine from young children, have the potential to lower the risk of MPI, and Royal College of Obstetricians and Gynaecologists guidance recommends offering this information to pregnant women (Khalil et al., 2025). Revello et al. evaluated the effectiveness of identifying HCMV-seronegative pregnant women and providing hygiene counseling in preventing MPI using a controlled trial design (Revello et al., 2015). In the prospective intervention group, HCMV-seronegative women received hygiene counseling at 11–12 weeks of gestation and consistent HCMV monitoring until delivery. The seroconversion rates were 1.2% and 7.6% in the intervention group and the control group, respectively.

#### 8.1.2 Vaccines

Efforts to develop a safe and effective HCMV vaccine have been ongoing since the 1970s, but no vaccine against HCMV has yet been approved for clinical use. Development of an effective HCMV vaccine faces multiple obstacles, including the complex biology of viral latency, reactivation and immune evasion, incompletely defined correlates of protective immunity, lack of consensus on the optimal target population for vaccination, and generally low public awareness of cCMV disease burden (Boppana et al., 2023). The prevalence of cCMV at birth and disease burden of cCMV are estimated to decrease within 20 years after the initiation of such an intervention (Bottino et al., 2023). On the basis of quality-adjusted life year and economic analyses, the Institute of Medicine of the U.S. National Academies ranked the vaccination of 12-year-old girls to prevent MPI during pregnancy a top priority for national vaccine development (Leruez-Ville et al., 2020). To date, numerous candidate vaccines have advanced to various phases of clinical development (Plotkin et al., 2020). In this review, findings from completed trials with published results are clearly distinguished from investigational candidates whose trials are ongoing and lack publicly available efficacy data (**Table 3**).

**Table 3:** Summary of HCMV vaccines.

Vaccine type	Core design/target	Representative vaccines	Latest progress (evidence cutoff: July 2025)	Advantages	Challenges	References
Live-attenuated vaccines & defective single-cycle infectious vaccines	Live-attenuated strains and defective single-cycle viral constructs	Towne–Toledo chimeric vaccine; V160 vaccine (based on the AD169 strain)	Phase I trials for both candidates completed	The Towne–Toledo chimeric vaccine exhibits favorable safety and tolerability when administered to healthy HCMV-seropositive individuals, without eliciting novel HCMV-specific immune responses.	The Towne–Toledo chimeric vaccine fails to induce pentameric complex-specific neutralizing antibodies. Three-dose V160 vaccinations can induce high-titer pentamer-specific neutralizing antibodies in all subjects; however, the overall protective efficacy is merely 42.4%.	(Adler et al., 2016; Li et al., 2021)
Adjuvanted recombinant protein vaccines	Targeting CMV glycoprotein B (gB) combined with adjuvants	The gB/MF59 vaccine (MF59 squalene adjuvant)	Phase II trials completed	High level gB specific antibody responses induce and enhance antibody dependent cellular phagocytosis (ADCP) responses.	Neutralizing antibody levels are low, with a vaccine efficacy of approximately 50% against primary infection, and this protective efficacy wanes within 15 months.	(Nelson et al., 2018; Boppana et al., 2023; Hu et al., 2024)
DNA vaccines	Plasmids encoding capsid protein pp65 and/or the envelope glycoprotein gB	VCL-CB01; ASP0113	Phase III trial in HSCT recipients completed	VCL-CB01 exhibits favorable safety profiles and potent immunogenicity; ASP0113 has elicited robust pp65-specific T-cell responses and late-phase gB-specific B-cell responses in phase II clinical trials.	ASP0113 failed to improve overall survival and reduce end-stage CMV-related diseases in HSCT populations.	(Wloch et al., 2008; Limaye et al., 2020)
mRNA vaccines	Nucleoside-modified mRNA encoding pp65, gB and pentameric complex (PC) encapsulated in lipid nanoparticles (LNPs)	mRNA-1647 (encoding pp65)	Phase II safety and immunogenicity trials completed; phase III randomized placebo-controlled trial (NCT05085366) ongoing	mRNA 1647 elicits durable HCMV specific IgG responses, induces high titre neutralizing antibodies in epithelial cells, and induces and enhances antibody dependent cell mediated cytotoxicity (ADCC).	If mRNA 1647 is intended for use in adolescents, longer term follow up is needed to confirm whether antibody levels can be sustained throughout the reproductive years.	(Hu et al., 2024)
Virus-like particle (VLP) vaccines	Enveloped virus-like particles (eVLPs) containing gB, gH, gL, pentameric complex and other glycoproteins	VBI-1501 (formulated with alum)	Phase I completed; higher dose phase II trials planned but not initiated	All doses show good safety; administration of 2 μg of aluminum phosphate-adjuvanted (APA) yields the highest levels of gB-specific antibodies and neutralizing antibodies with favorable tolerance	/	(Langley et al., 2024)
Viral vector vaccines	Recombinant attenuated heterologous viral vectors delivering CMV immunogens (gB, pentameric complex, T-cell antigens, etc.)	ALVAC-CMV (a canarypox vector expressing gB)	Phase I and phase II immunogenicity studies completed	Boosting with a live attenuated vaccine combination can elicit neutralizing antibody titers approaching those seen following natural infection.	Risk of pre-existing vector-specific immunity	(Bialas and Permar, 2016)
Peptide vaccines	Viral peptide antigens targeting CMV-specific T cells	CMVPepVax	Phase II trial in HSCT recipients completed	Stimulates epitope-specific T-cell responses with favorable safety; induces immune responses in 50% of subjects and CMV-specific T cells in 40% of pretransplant patients; prevents CMV-associated diseases in HSCT recipients	CMVPepVax can be administered only to HLA A*0201 recipients	(Nakamura et al., 2016)

Data on the incidence of MPI and MNPI in adolescent females from a prospective study by Paris et al. demonstrated that current diagnostic methods can effectively identify MPI and MNPI in this population (Paris et al., 2023). Therefore, Paris et al. recommended that both seropositive and seronegative adolescent females infected with HCMV be included in clinical trials to evaluate the efficacy of HCMV vaccines. Two completed phase II RCTs demonstrated that a recombinant glycoprotein B (gB) vaccine provided only moderate efficacy (approximately 50%) in reducing HCMV infection in seronegative women. However, this candidate did not advance to phase III efficacy trials, likely due to the waning of vaccine-induced immunity over time (Nelson et al., 2020). Moderna recently developed a messenger RNA (mRNA)-based HCMV vaccine, mRNA-1647, that encodes gB and the gH/gL/UL128/UL130/UL131A pentameric glycoprotein complex (pentamer) (Hu et al., 2024; Wu et al., 2024). This vaccine has successfully completed phase II trials for safety and immunogenicity and is currently undergoing a phase III randomized placebo-controlled trial in healthy individuals aged 16 to 40 years (Clinical Trial Number NCT05085366) (Pinninti and Boppana, 2023). However, a mathematical model designed by Byrne et al. to characterize the relationship between viral shedding in young children and maternal HCMV infection during pregnancy predicted that HCMV vaccines with moderate protective efficacy (40%–50%), such as the recombinant gB/MF59 vaccine and V160, a replication-deficient HCMV vaccine, might yield greater population-level benefit if administered to 2-month-old children compared with vaccination of women of child bearing age (Byrne et al., 2022). It is crucial to emphasize that this remains a simulation-based hypothesis; the predicted benefit has not yet been substantiated by prospective clinical trial data and therefore cannot be considered established clinical effectiveness. The concept of early childhood vaccination as a population-level prevention strategy necessitates further validation through real-world clinical studies.

### 8.2 Second-step prevention strategies

#### 8.2.1 Hyperimmune globulin

HCMV-specific, high-titer HIG has garnered significant interest for the prevention of cCMV, although findings have been inconsistent across studies of varying evidence levels. Early encouraging evidence emerged from non-randomized observational cohort studies, most notably the work by Nigro et al., which suggested that HIG could reduce both vertical transmission and symptomatic cCMV in fetuses exposed to intrauterine HCMV (Nigro et al., 2005). Nevertheless, this observational investigation is subject to well-recognized selection bias, and non-randomized study outcomes cannot establish causal therapeutic efficacy. Promising biological plausibility and observational signals do not equate to proven clinical benefit. In contrast, a high-quality, multicenter, double-blind RCT conducted by Hughes et al. demonstrated that the administration of HCMV HIG before 24 weeks of gestation in women with MPI did not reduce the incidence of cCMV infection, fetal death, or neonatal death among offspring (Hughes et al., 2021). As a continuation of this trial, a 2-year follow-up study to assess motor, cognitive, and hearing outcomes in infants; longer-term outcomes; and the severity of infection is underway. Recent pharmacokinetic studies have confirmed that the *in vivo* half-life of HIG is only 11 days, which necessitates repeated infusions (Leruez-Ville et al., 2020). Coste Mazeau et al. specifically investigated the potential of Cytotect CP^®^, an HIG product manufactured by Biotest (Germany), as a candidate agent for the prevention or treatment of cCMV infection using a first-trimester placental villus model (Coste Mazeau et al., 2022). Their results demonstrated that Cytotect CP^®^ shows potential for the prevention of cCMV infection, with favorable efficacy and low toxicity in different paths of infection. However, such preclinical and *ex vivo* experimental findings do not necessarily demonstrate clinical efficacy. To date, routine clinical administration of HIG has not been recommended.

#### 8.2.2 Valacyclovir

Valacyclovir is a prodrug of acyclovir that is synthesized by the addition of the naturally occurring amino acid L-valine. VCV inhibits HCMV DNA polymerase, subsequently reducing maternal HCMV loads at high dosages (8 g daily) (D'Antonio et al., 2023). In a meta-analysis, oral daily administration of 8 g of VCV could reduce HCMV vertical transmission in women with MPI during the periconceptional period or the first trimester of pregnancy, with a low rate of adverse events (Chatzakis et al., 2024). In a prospective, randomized, double-blind, placebo-controlled trial, pregnant women with MPI acquired during the periconceptional period or the first trimester of pregnancy received oral VCV or placebo until amniocentesis at 21 or 22 weeks of gestation (Shahar-Nissan et al., 2020). Shahar-Nissan et al. reported that the fetal infection rate decreased in the women with MPI during the first trimester, whereas no significant change in the fetal infection rate was observed among the women with periconceptional infection. This finding was based on the significantly earlier treatment of women with MPI acquired during the first trimester, closer to the time of maternal infection. A large nationwide Danish cohort study revealed that exposure to VCV during the first trimester of pregnancy was not associated with an increased risk of major birth defects (Pasternak and Hviid, 2010). Egloff et al. evaluated the efficacy of VCV on the basis of maternal VLs following HCMV seroconversion and reported that VCV was effective mainly in viremia-positive cases, reducing the maternal-fetal transmission rate by nearly 60%, but had no significant effect when viremia was undetectable (Egloff et al., 2023). Therefore, prompt initiation of VCV may be considered for women with periconceptional or first-trimester MPI to prevent maternal-fetal transmission. Notably, VCV has not received regulatory approval for this obstetric indication. Therefore, its prescription should be restricted to specialists, contingent upon an individualized risk-benefit assessment and the acquisition of adequate informed consent.

### 8.3 Third-step prevention strategies

Once fetal intrauterine HCMV infection is confirmed, antiviral therapy targeting the infected fetus constitutes tertiary prevention. A multicenter, open-label, single-arm phase II clinical trial—the first study reporting the efficacy of an antiviral drug to treat HCMV-infected fetuses—reported that administration of oral VCV at 8 g daily during pregnancy effectively improved outcomes in fetuses with moderately symptomatic HCMV infection (Byrne et al., 2022). Even if fetal infection has already occurred, VCV can effectively limit the HCMV load in the amniotic fluid and neonatal blood at birth (Egloff et al., 2023). Nevertheless, the use of VCV for treating proven fetal infection remains investigational and is not recommended for routine clinical practice. Additional RCTs are warranted to establish both its efficacy and long-term maternal-infant safety.

## 9 Discussion

With the accumulation of evidence confirming the effectiveness of early intervention for children with SNHL, continuous advances in antiviral therapy, and the recent development of candidate vaccines applicable to pregnant women and neonates, it is highly important to formulate effective strategies for population health education and disease prevention. Preventive strategies against HCMV infection focus on three core objectives: reducing the risks of MPI and reinfection, reducing the risk of mother-to-child transmission, and reducing the incidence of symptomatic cCMV infection (**Figure 1**).

**Figure 1:**
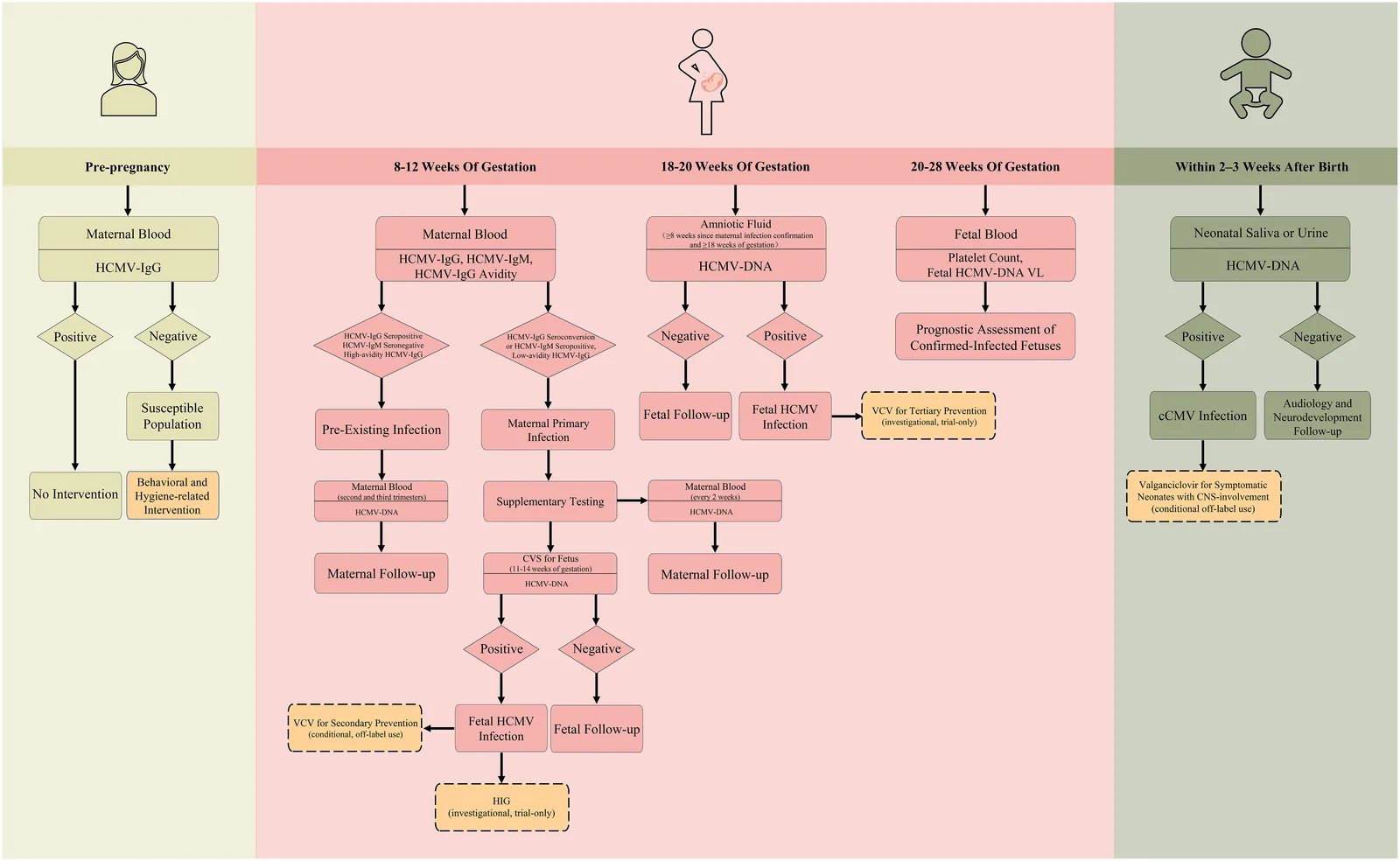
Strategies for the detection and prevention of HCMV. This algorithm represents a proposed framework based on the evidence reviewed; it does not represent a guideline-based algorithm. Solid borders: established clinical testing, sampling, diagnostic criteria, and consensus-supported routine follow-up. Dashed borders: conditional, off-label, or investigational approaches.

Given that close contact with toddlers is a well-established, high-risk exposure factor for HCMV infection, women of childbearing age should receive systematic health education on cCMV infection and relevant preventive measures as early as the preconception and gestational periods to avoid MPI or reinfection in early pregnancy. However, since universal routine first-trimester HCMV screening is not uniformly endorsed by international guidelines, local obstetric policies should be followed. The HCMV vaccine mRNA-1647 is currently undergoing a phase III randomized placebo-controlled clinical trial among healthy individuals aged 16 to 40 years. Nevertheless, the optimal target population for HCMV vaccination remains undetermined. Because human papillomavirus vaccination is routinely administered to children at 12 years of age, simultaneous HCMV vaccination can be considered in this age group (Boppana et al., 2023). However, if vaccination among 12-year-old girls is adopted to prevent cCMV infection, vaccines and immunization schedules to provide long-term protective immunity lasting 10 to 20 years are urgently needed.

In populations with extremely high HCMV seroprevalence, maternal reinfection with novel HCMV strains has been identified as an independent risk factor for the delivery of neonates with cCMV infection (Yamamoto et al., 2010). VCV confers several key advantages: oral administration and substantially lower cost with only mild adverse effects including thrombocytopenia, nausea, headache, and abdominal pain (Leruez-Ville et al., 2016). While existing evidence indicates that prompt preventive VCV therapy after first-trimester MPI may reduce vertical transmission risk, such intervention is not currently approved by the FDA for this indication and must be carried out under strict supervision.

The early identification of neonates with cCMV infection facilitates regular clinical follow-up and timely intervention, enabling the early detection and standardized management of neurodevelopmental disorders, including SNHL. Although off-label and lacking formal FDA approval, valganciclovir is indicated for moderately to severely symptomatic neonates with central nervous system involvement to preserve auditory and neurodevelopmental outcomes (Leruez-Ville et al., 2024). Regardless of whether antiviral interventions are implemented during pregnancy or the neonatal period, children with cCMV infection require regular audiological and developmental assessments and prompt supportive treatment to optimize their long-term clinical prognosis.

Despite accumulating evidence regarding HCMV diagnosis and prevention in pregnancy and neonates, significant limitations and uncertainties persist within the current literature. These factors warrant careful consideration when translating research findings into clinical practice. First, maternal HCMV screening policies and diagnostic test performance exhibit substantial heterogeneity. International guidelines do not support universal first-trimester screening; instead, practices vary widely, ranging from targeted high-risk screening to the absence of formal population-level programs. Furthermore, assay performance is highly dependent on the platform, specimen collection timing, and study population. Serological assays show marked discrepancies between CLIA and ELISA platforms, and IgG avidity testing proves unreliable when HCMV IgG concentrations are low. Molecular assays also demonstrate variable performance: DBS PCR sensitivity ranges widely (34.4%–100%) across cohorts, and a negative result cannot rule out cCMV infection. Even the reference standard, amniotic fluid HCMV DNA PCR, carries a 5%–10% false-negative rate, primarily due to an insufficient interval between maternal infection and amniocentesis. No single laboratory biomarker can independently confirm or exclude maternal primary HCMV infection, making multiparameter combined interpretation mandatory.

Second, the evidence supporting various preventive and therapeutic interventions remains limited or conflicting. Although HIG demonstrated promising signals in early non-randomized observational studies, high-quality, multicenter, double-blind RCTs have failed to confirm its clinical benefit in preventing intrauterine transmission; consequently, HIG remains an investigational measure restricted to clinical trials. For VCV, secondary prevention (aimed at reducing vertical transmission risk in maternal primary infection prior to confirmed fetal infection) is supported by RCT and meta-analytic evidence. However, this remains an off-label application that necessitates careful risk-benefit evaluation and informed consent. Conversely, VCV for tertiary prevention (treating confirmed intrauterine fetal infection) lacks RCT validation, being supported only by a single-arm Phase II trial, and is therefore not recommended for routine clinical practice. Regarding cellular immunity assays such as IGRAs, while they have shown predictive value in transplant cohorts, their optimal cut-off values and clinical utility in pregnant populations remain poorly established. Finally, novel point-of-care tests, such as the MIRA LFD assay for detecting HCMV in breast milk, have been validated solely in laboratory settings and lack large-scale, real-world clinical validation.

Third, many observational studies are limited by potential confounding factors, heterogeneous case definitions, variable follow-up durations, and inconsistent inclusion criteria. A substantial proportion of existing data originates from high-income cohorts; consequently, the generalizability of these findings to low- and middle-income settings, which often have distinct seroprevalence profiles, laboratory infrastructure, and obstetric care pathways, remains unclear. Furthermore, long-term neurodevelopmental follow-up data for many interventions, including prenatal antiviral exposure, remain incomplete.

Collectively, core diagnostic workflows are well supported by evidence, including serial maternal serology for MPI, timely amniotic fluid PCR for prenatal diagnosis, and neonatal saliva or urine PCR within 2–3 weeks of birth for definitive cCMV confirmation. Conversely, many screening strategies, prognostic biomarkers, and antenatal therapies remain uncertain. Further well-designed, prospective, and ideally randomized clinical trials are necessary before their widespread clinical adoption.
